# Altered DNA methylation of *CYP2E1* gene in schizophrenia patients with tardive dyskinesia

**DOI:** 10.1186/s12920-022-01404-8

**Published:** 2022-12-09

**Authors:** Ping Zhang, Yanli Li, Kesheng Wang, Junchao Huang, Brenda Bin Su, Chun Xu, Zhiren Wang, Shuping Tan, Fude Yang, Yunlong Tan

**Affiliations:** 1 Beijing HuiLongGuan Hospital, Peking University HuiLongGuan Clinical Medical School, Beijing, 100096 China; 2 Department of Family and Community Health, Robert C. Byrd Health Sciences Center, School of Nursing, West Virginia University, Morgantown, WV 26506 USA; 3 Department of Health and Biomedical Sciences, College of Health Affairs, University of Texas Rio Grande Valle, Brownsville, TX USA

**Keywords:** Schizophrenia, Tardive dyskinesia, Pyrosequencing, DNA methylation, *CYP2E1*, Oxidative stress

## Abstract

**Background:**

About 20–30% of patients with schizophrenia develop tardive dyskinesia (TD). Oxidative stress is one potential causes of TD. *CYP2E1* is considered as an oxidative stress-related gene, however, no study has been reported on the DNA methylation levels of the *CYP2E1* in schizophrenia or TD.

**Methods:**

A total of 35 schizophrenia patients with TD, 35 schizophrenia patients without TD (NTD), and 35 health controls (HCs) were collected in Beijing, China. DNA was extracted from peripheral blood samples. The promoter methylation levels of *CYP2E1* were detected using pyrosequencing. The generalized linear model (GLM) was used to examine the methylation levels of three CpG sites among three diagnostic groups (TD vs. NTD vs. HC).

**Results:**

The average methylation levels were 8.8 ± 10.0, 14.5 ± 11.9 and 15.1 ± 11.3 in TD, NTD and HC groups, respectively. The *F*-test in GLM revealed overall differences in the average of methylation levels of three CpG sites among three diagnostic groups (*p* = 0.0227) and in the third CpG site (*p* = 0.0026). Furthermore, the TD group had lower average methylation levels than HC and NTD groups (*p* = 0.0115 and 0.0268, respectively). Specifically, TD group showed lower methylation levels in the third CpG site than HC and NTD groups (*p* = 0.0012 and 0.0072, respectively). Additionally, associations of the methylation levels with clinical features in the TD group were observed using Spearman correlation analysis.

**Conclusion:**

This study provides the first evidence of DNA methylation levels in the promoter of *CYP2E1* gene associated with schizophrenia and TD. The abnormal DNA methylation might serve as a potential mechanism for TD.

## Background

Tardive dyskinesia (TD), with a prevalence of 20–30% in schizophrenia (SCZ) patients and up to 50% in SCZ patients > 50 years old, is characterized by repetitive, involuntary movements of the extremities or trunk and is considered as one of the serious adverse effects of long-term antipsychotic medications for SCZ and other mental disorders [1–4]. TD is potentially irreversible and linked with poor quality of life and increased medical co-morbidity and mortality [5]. Furthermore, TD is a frequent disorder that can occur in early stages of SCZ and intensifies with increasing age and duration of illness, even in patients treated with atypical antipsychotics. Despite a considerable number of related research have been conducted, the pathogenesis of TD remains poorly understood [6].

Oxidative stress is a state when there is an imbalance between the antioxidant defense system and pro-oxidant process in favor of the latter and it is one of the potential causes of TD [7, 8]. One hypothesis based on both in vivo and in vitro studies of oxidative stress pointed out that neurotoxic free radical production is likely a consequence of antipsychotic medication and might result in the occurrence of TD [9]. Recently, mounting evidence suggested the role of disturbance of antioxidant defense system and presence of oxidative stress via the biochemical mechanisms underlying TD [10–12]. Ala9Val polymorphism of the *MnSOD* gene has been found to be associated with TD [13].

In recent years, researchers have proposed that genetic predisposition, environment and epigenetic processes and their interactions may play important roles in the development of psychiatric disorders, especially in SCZ and TD [2, 3, 14–16]. DNA methylation is an important epigenetic modification involving the addition of a methyl group at the 5th carbon of cytosines preceding guanines (CpG dinucleotides) and may play a role in regulating gene expression when implicated in SCZ [17–20]. It has been shown that pyrosequencing can quantify DNA methylation levels of single CpG site [21–23], which has been used to detect methylation levels in SCZ [24–28] and TD [29].

The *CYP2E1* (also known as *CPE1*; *CYP2E*; *P450-J*; *P450C2E*) is located at 10q26.3 [30] and involved in drug metabolism and synthesis of cholesterol, steroids and other lipids, and is associated with gluconeogenesis, cancers, liver conditions, diabetes, SCZ, Parkinson’s disease, cognitive and neurobiological phenotypes [31–38]. Furthermore, the CYP2E1 level is related to oxidative stress and mitochondrial dysfunction, especially, enhanced CYP2E1 activity could further facilitate the formation of potentially toxic metabolites, leading to more severe oxidative stress and mitochondrial dysfunction [39]. Moreover, it has been reported that the *CYP2E1* is an oxidative stress-related gene in several pathophysiological conditions including obesity, diabetes, and metabolism [39–42]. To our knowledge, no study has analyzed the DNA methylation levels of the *CYP2E1* using pyrosequencing in SCZ or TD. The purpose of the current study was to examine whether the promoter methylation levels of the *CYP2E1* were altered in SCZ patients and TD. In addition, we sought to explore the relationships between the promoter methylation levels of *CYP2E1* gene and clinical features in SCZ patients and TD.

## Methods

### Subjects

Totally, 35 SCZ patients with TD (TD group) and 35 SCZ patients without TD (NTD group) meeting DSM-IV criteria for SCZ were recruited from Beijing HuiLongGuan Hospital, Beijing, China. Clinical diagnosis of TD was confirmed by two experienced psychiatrists using the criteria of Schooler and Kane (1982) [43]. The severity of TD symptoms was rated by two experienced investigators using the Abnormal Involuntary Movements Scale (AIMS), with an inter-rater correlation coefficient (ICC) greater than 0.80. The patients’ psychotic symptoms were assessed using the Positive and Negative Syndrome Scale (PANSS) [44] with an ICC greater than 0.85 maintained for the PANSS total score after the scale training. Individuals in the TD group had the Abnormal Involuntary Movement Scale (AIMS) scored higher than 3 in at least one part or at least 2 in two or more parts. The same criteria were used for NTD group except that AIMS = 0. At the same period, 35 age-, sex- and education-matched health controls (HC group) assessed by the same investigators were also enrolled from the local community. Data on duration of disease, current medication time and medication types were also collected for further statistical analysis.

### Genomic DNA extraction, bisulfite treatment and pyrosequencing

Fasting venous blood from forearm vein was obtained from each participant at 7:00 am in the next morning after the day of clinical assessment. DNA was extracted from peripheral blood samples using a standard genomic DNA extraction kit (QIAGEN, Germany). The purified DNA was processed with DNA bisulfite conversion kit (TIANGEN BIOTECH Co., Ltd., Beijing, China). Then PCR assays were performed to amplify parts of the CpG islands in the promoter region within the *CYP2E1* gene. The PCR temperature was 95℃ for denaturation, 60℃ for renaturation, and 20℃ for termination. Final components of PCR reagents included 34.8ul water, 10ul 5×buffer (KAPA), 1ul dNTP (10 mM/each), 1ul primer (up 50 pM/ul), 1ul primer (down 50 pM/ul), 2ul template, and 0.2ul Taq (5U/ul). Generally, DNA fragments were amplified using the PyroMark PCR Kit (QIAGEN, Germany) from 2 µl bisulfite-treated genomic DNA sample. Sample preparation and pyrosequencing reaction were then carried out using the PyroMark Q96 ID (QIAGEN, Germany). For pyrosequencing, we used the PyroMark Assay Design 2.0 software to design primers for the analysis of *CYP2E1*. With the relative best primer designed by PyroMark Assay Design 2.0 software, we obtained and evaluated the methylation levels of three of them in the PCR product. The three CpG sites are located at position, chr10:135342782–135,342,814 (based on Human GRCh37/hg19 assembly). The forward primer sequence was 5′-AGGGGGAAGAGATTTATTGAAA-3′, the reverse one was 3′-ACCCAAAAAAAAATAAAAACTTCCATAT-5′, and the probe sequencing was TTAGGGAGAGGAGGG (Table 1). Electrophoresis detection with Gel Imaging System 1600 (Tanon Science & Technology Co., Ltd., Shanghai, China) was used to ensure the accuracy and specificity of the PCR product. The methylation status of each site was automatically analyzed with the Pyro Q-CpG software and the percentage of methylation (5%-mC) at each CpG site was calculated as a ratio of the methylated signal intensity to the sum of both methylated and unmethylated signals after background subtraction, ranging from 0 (completely unmethylated) to 1 (completely methylated). Quantitative methylation results were considered both as percentage of individual CpG sites and as average of the methylation percentage of the three investigated CpG sites. All individual sample passed quality control (the total amount of DNA more than 1ug, the OD260/OD280 ratio between 1.7 and 1.9, and single DNA band); therefore, the analysis was based on 35 SCZ with TD, 35 SCZ without TD and 35 controls.

**Table 1:** Primer sequences used in the pyrosequencing analysis

Primer type	Primer sequence	CpG sites	Position 5′ − 3′
Forward	5′- AGGGGGAAGAGATTTATTGAAA-3′	3	chr10:135342648–135,342,669
Reverse	3′- ACCCAAAAAAAAATAAAAACTTCCATAT- 5′		chr10:135342997–135,342,970
Sequencing	5′- TTAGGGAGAGGAGGG-3′		chr10:135342782–135,342,814

### Statistical analysis

The categorical variables were presented in their raw values and continuous variables were presented in the form of mean ± standard deviation (SD). The chi-square (*χ*^*2*^) test was used to analyze sex across TD, NTD and HC groups. The *F-*/*t-*test in a generalized linear model (GLM) was used to compare means of continuous variables between diagnostic groups.

The normality of DNA methylation levels was tested by the SAS PROC UNIVARIATE. Because the methylation data is percentage, we used arcsine transformation-one of the most common methods for transforming percent, proportions, and probabilities [45]. The association between potential predictors and transformed methylation levels were detected using SAS PROC GLM. The differences in the transformed methylation levels among the TD, NTD and HC diagnostic groups for each CpG site and the average of the methylation percentage of three CpGs sites were compared using the *F-*/*t-*test in GLM. The *F-*test in the GLM was used to detect the overall significance among three groups, while the *t-*test was applied to assess difference between two groups. To deal with the multiple testing problem, Bonferroni correction was used for statistical significance. Considering three CpG sites, the Bonferroni corrected significant level will be a *p* value < 0.05/3 = 0.0167.

Spearman’s rank correlation analyses were performed to explore the relationship among methylation levels of each CpG site, the average methylation level of three CpG sites, and clinical features in TD and NTD groups.

All statistical analyses were performed with SAS version 9.4 (SAS Institute, Cary, NC, USA).

## Results

### Demographic and clinical characteristics

There were no significant differences in age, education, and sex among HC, NTD and TD groups (all *p* values > 0.05) (Table 2). SCZ patients with and without TD did not differ in duration of disease, current medication time and medication types (both *p* values > 0.05). Compared to the NTD group, the TD group had significantly higher PANSS negative and PANSS total scores (*p* = 0.012 and 0.037, respectively) (Table 2).

**Table 2:** Demographic and clinical characteristics of health controls and schizophrenia patients with and without tardive dyskinesia

Variables	HC group (*n* = 35)	NTD group (*n* = 35)	TD group (*n* = 35)	*F* / *χ*^*2*^ / *t*	*p*
Age (years)	44.4 ± 11.6^a^	44.9 ± 11.3	45.1 ± 12.3	0.03	0.968
Education (years)	12.0 ± 3.1	11.4 ± 2.9	10.3 ± 2.7	3.09	0.051
Sex (male/female)	20/15	20/15	20/15	0	1.000
*PANSS score* Total (PANSST)	–	67.1 ± 16.0	75.6 ± 16.2	2.13	0.037
Positive (PANSSP)	–	17.5 ± 6.8	19.4 ± 6.1	1.19	0.240
Negative (PANSSN)	–	17.8 ± 6.5	22.2 ± 7.0	2.60	0.012
General psychopathology (PANSSG)	–	30.6 ± 8.4	33.9 ± 7.0	1.75	0.086
AIMS total score	–	–	14.6 ± 5.8	–	–
Duration of disease (years)		21.5 ± 12.1	21.1 ± 11.3	− 0.14	0.887
Current medication time (months)	–	30 (98)	30 (132)	− 0.79	0.428
Medication types (atypical/typical antipsychotics)	–	30/5	32/3	0.57	0.452

*AIMS* Abnormal involuntary movement scale;* HC* Health controls;* TD* Tardive dyskinesia;* NTD* Without tardive dyskinesia;* PANSS* Positive and negative syndrome scale

^a^ indicates mean ± SD for continuous variables; χ^2^ value is based on the chi-square test; *F* value is based on the generalized linear model for comparing three groups; *t* value is based on the generalized linear model for comparing two groups

### Comparison of methylation levels among three diagnostic groups

The mean values of three CpG sites (5′−3′ chr10:135342782–13,542,783, 135,342,800–135,342,801, and 135,342,812–135,342,813) were 8.8 ± 10.0, 14.5 ± 11.9 and 15.1 ± 11.3 for TD, NTD and HC groups, respectively (Table 3). After arcsine transformation, the DNA methylation levels were close to normal distribution based on the Kolmogorov-Smirnov statistics. The skewness values for three CpG sites were − 0.439, − 0.118, and − 0.041, respectively and kurtosis values were = − 0.781, − 1.415, − 1.534, respectively. These values are within (− 2, 2) indicating normal distribution. The Levene *F* Statistics showed homogeneity of the variance for three CpG sites and the average of the three CpG sites among three groups (all four *p* values are > 0.05). Then the linear GLM was used to detect associations of potential factors with methylation levels.

**Table 3:** Generalized linear model analysis of DNA methylation levels of three CpG sites in promoter of the *CYP2E1* gene

Group	Parameters	Site 1	Site 2	Site 3	Average
TD	Mean ± SD	11.1 ± 10.6	8.7 ± 10.8	6.6 ± 9.6	8.8 ± 10.0
NTD		16.4 ± 12.3	14.3 ± 12.5	12.7 ± 11.0	14.5 ± 11.9
HC		15.9 ± 11.3	15.5 ± 12.1	13.8 ± 10.9	15.1 ± 11.3
Levene’s *F* Statistics	*F*	0.045	0.316	0.559	0.199
	*p*	0.956	0.730	0.574	0.920
TD vs. NTD vs. HC	*F*	2.64	3.79	6.23	3.93
	*p*	0.0762	0.0258	0.0026*	0.0227
TD vs. HC	*t*	− 1.89	− 2.58	− 3.33	− 2.57
	*p*	0.0615	0.0114*	0.0012*	0.0115*
NTD vs. HC	*t*	0.19	− 0.45	− 0.59	− 0.33
	*p*	0.8531	0.0657	0.5561	0.7442
TD vs. NTD	*t*	− 2.08	− 2.13	− 2.74	− 2.25
	*p*	0.0404	0.0355	0.0072*	0.0268

Abbreviations: TD, tardive dyskinesia; NTD, without tardive dyskinesia; HC, health controls; *F* value is based on the generalized linear model for comparing three groups; *t* value is based on the generalized linear model for comparing two groups. * p < 0.0167 based on Bonferroni correction

**Fig. 1:**
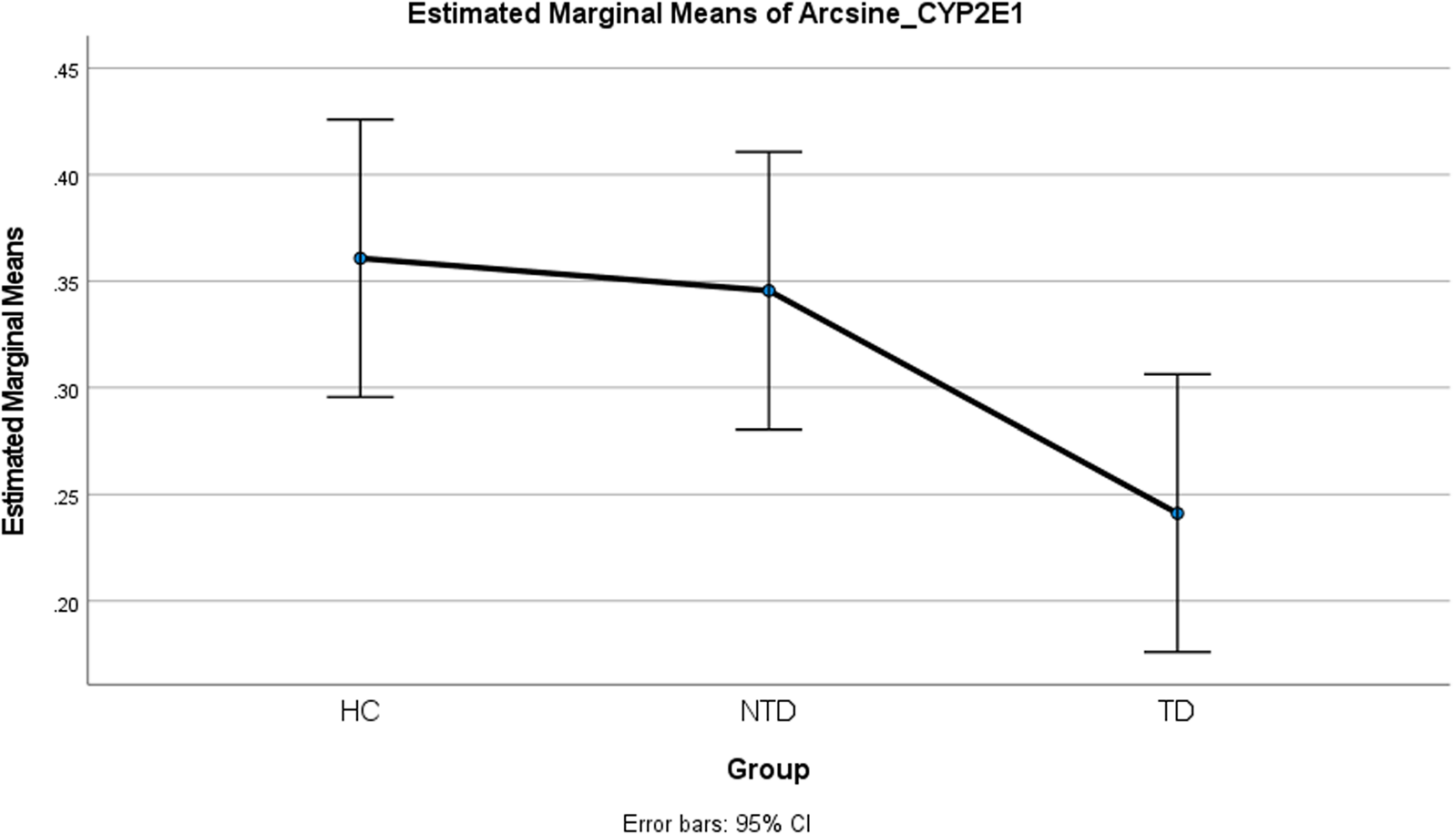
Marginal means plot of arcsine transformed average methylation of three CpG sites in *CYP2E1* gene for comparing three groups. TD, tardive dyskinesia; NTD, without tardive dyskinesia; HC, health controls

The results of the differences in the transformed methylation levels among the TD, NTD and HC groups for each CpG site and the average of the methylation percentage of three CpG sites were presented in Table 3. The *F-*test revealed that three groups had significant differences in DNA methylation level for the second and the third CpG site and the average DNA methylation level of three CpG sites (*p* = 0.0258, 0.0026 and 0.0227, respectively) (Table 3; Fig. 1). Furthermore, the *t-*test showed TD group had lower methylation levels in each CpG site and in the average of three CpG sites comparing with NTD group (*p =* 0.0404, 0.0355, 0.0072, and 0.0268, respectively); while TD had lower methylation levels in the second and the third CpG sites and the average of three CpG sites comparing with HC group (*p =* 0.0114, 0.0012, and 0.0115, respectively). *P* values smaller than 0.0167 (indicated with a “*”) show statistical significance based on Bonferroni correction in Table 3.

### Correlation between methylation level and clinical features

Table 4 showed that the methylation level in the second CpG site had borderline correlation with AIMS score in the TD group (*p ≤* 0.05). In addition, the average of three CpG sites had borderline correlations with duration of disease in the TD group (*p ≤* 0.05).

**Table 4:** Spearman correlation coefficients among methylation levels and clinical characters for TD (above diagonal) and NTD (below diagonal)

Variable	CYP2E1 site 1	CYP2E1 site 2	CYP2E1 site 3	CYP2E1 average	Age	Duration	PANSST	PANSSP	PANSSN	PANSSG	AIMS
CYP2E1 site 1	1.000	0.740***	0.809***	0.908***	− 0.042	0.158	− 0.016	− 0.074	− 0.053	0.113	0.070
CYP2E1 site 2	0.955***	1.000	0.785***	0.910***	0.105	0.216	− 0.025	− 0.085	− 0.030	0.126	0.294*
CYP2E1 site 3	0.983***	0.970***	1.000	0.851***	0.022	0.135	− 0.004	− 0.114	− 0.092	0.224	0.233
CYP2E1 average	0.993***	0.977***	0.987***	1.000	0.121	0.294*	− 0.040	− 0.067	− 0.096	0.128	0.263
Age	0.141	0.031	0.126	0.106	1.000	0.776***	− 0.027	0.128	− 0.080	− 0.083	0.265
Duration	0.178	0.056	0.152	0.142	0.884***	1.000	0.073	0.132	0.069	− 0.073	0.173
PANSST	− 0.061	− 0.024	− 0.006	− 0.062	− 0.162	− 0.152	1.000	0.832***	0.764***	0.796***	− 0.227
PANSSP	0.231	0.184	0.233	0.219	0.046	− 0.152	0.703***	1.000	0.455*	0.609**	− 0.223
PANSSN	− 0.238	− 0.170	− 0.193	− 0.225	− 0.215	− 0.247	0.641***	0.192	1.000	0.384*	− 0.156
PANSSG	− 0.050	0.013	0.012	− 0.031	− 0.298*	− 0.308*	0.711***	0.479**	0.264	1.000	− 0.029
AIMS	–	–	–	–	–	–	–	–	–	–	1.000

PANSS refers to positive and negative syndrome scale; PANSST refers to the total score; PANSSP refers to positive score; PANSN refers to negative score; PANSSG refers to the general psychopathology score; AIMS refers to abnormal involuntary movement scale; AIMSS refers to AIMS score. * refers to *p ≤* 0.05, ** refers to *p* < 0.01, *** refers to *p* < 0.001 in Spearman correlation analysis

## Discussion

In this study, we analyzed promoter methylation levels of *CYP2E1* involved in oxidative stress in three diagnostic groups, TD, NTD and HC. There were overall differences in the average of methylation levels of three CpG sites of the *CYP2E1* among three groups. Furthermore, the TD group showed that average methylation levels was lower than HC and NTD groups. Specifically, TD showed much lower methylation levels at the third CpG site than HC and NTD groups.

In the oxidative stress hypothesis for the pathogenesis of TD, it has been proposed that long-term neuroleptic exposure and dopamine receptor blockade could increase free radical generation and lipid peroxidation (*LPO*) through elevating dopamine turnover and augmenting monooxygenases activity [7]. Among all kinds of monooxygenases, the *CYP2E1* encoding cytochrome P4502E1 monooxygenase is responsible for metabolizing a broad range of small, hydrophobic substrates and drugs [46, 47]. Notably, CYP2E1 may generate reactive oxygen metabolites (ROM) by oxidation during the metabolism of those exogenous and endogenous compounds [48]. Additionally, CYP2E1 undergoes “uncoupling” of its catalytic cycle wherein electrons are consumed to generate reactive oxygen species (ROS). To protect the organism against ROM and ROS, a variety of enzymatic and nonenzymatic mechanisms have evolved [49]. Oxidative stress occurs when the equilibrium of the oxidant/antioxidant balance is disrupted and tilts toward the former, which is usually accompanied with harmful effects to cell survival including *LPO* and oxidative modification of proteins and nucleic acids [50]. Mitochondria and mitochondrial components such as DNA are particularly vulnerable to CYP2E1 induced oxidative stress, and that have been reported in multiple pathophysiological conditions, including obesity, diabetes and non-alcoholic steatohepatitis [39]. Nevertheless, relatively few studies of DNA methylation have been published on psychiatric disorders, specifically on SCZ and TD. In the present study, we found significantly decreased DNA methylation levels at the promoter of the *CYP2E1* gene in SCZ patients with TD. Previous study has shown that promoter methylation has been shown to be inversely correlated with transcription activity and gene expression [35, 51, 52]. Accordingly, we hypothesized that, SCZ patients with TD might have higher transcriptional activity and translational level of *CYP2E1* compared to SCZ patients without TD and health controls. It has been reported that CYP2E1 plays an important role in oxidative stress and mitochondrial dysfunction [39]. Obviously, this is consistent with the aforementioned oxidative stress hypothesis for the pathogenesis of TD.

The *CYP2E1* has been suggested to be associated with drug metabolism and cancers, liver conditions, diabetes, SCZ, Parkinson’s disease, cognitive and neurobiological phenotypes [31–38, 53, 54]. Previously, one Japanese case-control study did not find linkage of the *CYP2E1 c1/c2* polymorphism to SCZ [55]; whereas one Chinese case-control study revealed that *CYP2E1* polymorphisms (SNP rs8192766 and rs2070673) were associated with susceptibility to SCZ [34]. Furthermore, Kaut et al. (2012) reported a decreased methylation of *CYP2E1* and increased expression of *CYP2E1* messenger RNA in patients with Parkinson’s disease, suggesting that epigenetic variants of this cytochrome contribute to Parkinson’s disease susceptibility [35]. Catanzaro et al. (2012) demonstrated associations between certain *CYP2E1* VNTR genotypes and drinking and/or smoking habits. Accordingly, they hypothesized that the A1/A1 VNTR genotype of *CYP2E1* gene may have a protective role against drinking- and/or smoking related cancers, and that A4/A4 of *CYP2E1-*VNTR may be a high-risk genotype during the early stages of cancer [33]. Recently, the methylation at *CYP2E1* was found to be associated with both autism spectrum disorder and expression differences in brain [56]. To the best of our knowledge, our present study provided the first evidence about the epigenetics of *CYP2E1* gene regarding the link to TD, a neurological disease.

Moreover, several studies have suggested that genetics, environment, and epigenetics may interact each other in the development of TD [2, 3, 13, 15]. However, the DNA methylation study in TD is still in its infancy [29, 57]. The present study focused on one oxidative stress-related gene - *CYP2E1* and provided the first evidence of altered methylation levels in the promoter of this gene involved in the pathogenesis of TD. Interestingly, Naselli et al. examined the polymorphisms and methylation of *CYP2E1* in correlation to its expression in both tumor and non-neoplastic liver cell lines [36]. They found that reduced DNA methylation, assessed both at genomic and gene level, was not consistently associated with the increase of enzyme expression; however, A2 and A3 *CYP2E1* alleles played a more important role in the expression of the enzyme. Furthermore, they found that both untreated tumor cell lines and Chang liver cells showed a hypermethylation of the two sites in DNA methylation analysis [36]. It will be necessary and promising to study the complex roles of genetic polymorphisms, gene expression and epigenetics of *CYP2E1* in SCZ and TD.

However, there are several limitations need to be stated. First, DNA methylation is organ or tissue-specific, the sample here we collected was peripheral blood, mainly due to the following two considerations: the difficulty in obtaining brain tissue for central nervous system disorders and the feasibility of biomarker research conduction. Blood samples may not provide reliable results and therefore, current results need further confirmation and validation. However, significant correlation in DNA methylation levels was found between the peripheral tissues and brain tissues for *CYP2E1* (rho = 0.8451) [58]. Furthermore, dysmethylation of *CYP2E1* was observed in Parkinson’s diseased brains [54]. Second, our sample size was relatively small to moderate. We used PROC POWER in SAS 9.4 to compute power for the three independent groups based on one-way ANOVA [59]. Based on the sample size of 35 individuals for each group, the power to detect the difference among three means of three CpG sites could reach 64% and the power could reach 78% for the third CpG site. Third, the methylation level differences between groups are very small. Therefore, our findings still need to be replicated in the future studies with larger groups of patients. Forth, age and education may influence the methylation levels though we did not find the associations of these covariates (age and education) with DNA methylation levels in the current study. Fifth, factors that may affect gene methylation level (such as age, medication, etc.) have been taken into account in present research, but they were not so specific. Hence, the confounding factors mentioned above need to be controlled more strictly in future studies. Besides, to explore the concrete effect of a relative small DNA methylation drop in the TD group on the expression of *CYP2E1* and to better understand the mechanisms of *CYP2E1* in TD, functional analysis such as gene expression are absolutely necessary to be involved in the future study. Additionally, there are potential genetic effects influencing the methylation level of *CYP2E1*, hence, information about family history of mental illness, and ideally, the methylation levels of other family members should also be measured in future studies. Last but not least, we did not genotype *CYP2E1* in our studied subjects, thus no correlation was conducted between *CYP2E1* genotypes and DNA methylation level. In future studies, the possibility that polymorphisms are also necessarily to be determined to better clarify the role of this gene in the pathology. After all, the DNA methylation of a gene it is not “per se” but oriented to predict gene expression and its regulation.

## Conclusion

Our study revealed that the DNA methylation level of the *CYP2E1* in TD group was significantly lower compared with the NTD and HC groups. Abnormal methylation of oxidative stress-related *CYP2E1* may well be associated with the susceptibility of TD. Furthermore, the methylation level in the second CpG site had borderline correlation with AIMS score in the TD group, while the average of three CpG sites had borderline correlation with duration of disease in the TD group. These findings may serve as a resource for replication in other ethnic populations. Future functional studies of this gene such as gene expression, a quantitative PCR analysis may help to better characterize the genetic architecture of TD.

## Data Availability

Pyrosequencing data are available at NCBI BioProject with Accession Number: PRJNA589914. https://www.ncbi.nlm.nih.gov/bioproject/?term=PRJNA589914. The direct web link of the excel data: https://submit.ncbi.nlm.nih.gov/ft/byid/wxh3eqct/pyroseq_data.csv.

## Abbreviations

AIMS: Abnormal involuntary movement scale
GLM: Generalized linear model
HC: Health controls
ICC: Inter-rater correlation coefficient
PANSS: Positive and negative syndrome scale
PANSST: Total score of PANSS
PANSSP: Positive score of PANSS
PANSN: Negative score of PANSS
PANSSG: General psychopathology score of PANSS
SCZ: Schizophrenia
TD: Tardive dyskinesia

## References

[1] Tarsy D, Lungu C, Baldessarini RJ (2011). Epidemiology of tardive dyskinesia before and during the era of modern antipsychotic drugs. J Handb Clin Neurol.

[2] Lanning RK, Zai CC, Muller DJ (2016). Pharmacogenetics of tardive dyskinesia: an updated review of the literature. Pharmacogenomics.

[3] Correll CU (2017). Epidemiology and Prevention of Tardive Dyskinesia. J Clin Psychiatry.

[4] Voelker R (2017). Tardive Dyskinesia drug approved. JAMA.

[5] Morrow T (2018). Two new drugs for Tardive Dyskinesia hit the market. Manag Care.

[6] Stegmayer K, Walther S, van Harten P (2018). Tardive Dyskinesia Associated with atypical antipsychotics: prevalence, Mechanisms and Management Strategies. CNS Drugs.

[7] Tsai G, Goff DC, Chang RW, Flood J, Baer L, Coyle JT (1998). Markers of glutamatergic neurotransmission and oxidative stress associated with tardive dyskinesia. Am J Psychiatry.

[8] Szota AM, Scheel-Krüger J (2020). The role of glutamate receptors and their interactions with dopamine and other neurotransmitters in the development of tardive dyskinesia: preclinical and clinical results. Behav Pharmacol.

[9] Cho CH, Lee HJ (2013). Oxidative stress and tardive dyskinesia: pharmacogenetic evidence. Prog Neuropsychopharmacol Biol Psychiatry.

[10] Zhang XY, Yao JK (2013). Oxidative stress and therapeutic implications in psychiatric disorders. Prog Neuropsychopharmacol Biol Psychiatry.

[11] Cadet JL, Lohr JB (1989). Possible involvement of free radicals in neuroleptic-induced movement disorders. Evidence from treatment of tardive dyskinesia with vitamin E. Ann N Y Acad Sci.

[12] Elkashef AM, Wyatt RJ (1999). Tardive dyskinesia: possible involvement of free radicals and treatment with vitamin E. Schizophr Bull.

[13] Lee HJ, Kang SG (2011). Genetics of tardive dyskinesia. Int Rev Neurobiol.

[14] Carroll LS, Owen MJ (2009). Genetic overlap between autism, schizophrenia and bipolar disorder. Genome Med.

[15] Csoka AB, Szyf M (2009). Epigenetic side-effects of common pharmaceuticals: a potential new field in medicine and pharmacology. Med Hypotheses.

[16] Gejman PV, Sanders AR, Duan J (2010). The role of genetics in the etiology of schizophrenia. Psychiatr Clin North Am.

[17] Wockner LF, Noble EP, Lawford BR, Young RM, Morris CP, Whitehall VL (2014). Genome-wide DNA methylation analysis of human brain tissue from schizophrenia patients. Transl Psychiatry.

[18] Hannon E, Dempster E, Viana J, Burrage J, Smith AR, Macdonald R (2016). An integrated genetic-epigenetic analysis of schizophrenia: evidence for co-localization of genetic associations and differential DNA methylation. Genome Biol.

[19] Lee SA, Huang KC (2016). Epigenetic profiling of human brain differential DNA methylation networks in schizophrenia. BMC Med Genomics.

[20] Pries LK, Gülöksüz S, Kenis G (2017). DNA methylation in Schizophrenia. Adv Exp Med Biol.

[21] Mikeska T, Felsberg J, Hewitt CA, Dobrovic A (2011). Analysing DNA methylation using bisulphite pyrosequencing. Methods Mol Biol.

[22] Fakruddin M, Chowdhury A (2012). Pyrosequencing-An alternative to traditional Sanger sequencing. Am J Biochem Biotech.

[23] Delaney C, Garg SK, Yung R (2015). Analysis of DNA methylation by pyrosequencing. Methods Mol Biol.

[24] Ikegame T, Bundo M, Sunaga F, Asai T, Nishimura F, Yoshikawa A (2013). DNA methylation analysis of BDNF gene promoters in peripheral blood cells of schizophrenia patients. Neurosci Res.

[25] Lott SA, Burghardt PR, Burghardt KJ, Bly MJ, Grove TB, Ellingrod VL (2013). The influence of metabolic syndrome, physical activity and genotype on catechol-O-methyl transferase promoter-region methylation in schizophrenia. Pharmacogenomics J.

[26] Gao S, Cheng J, Li G, Sun T, Xu Y, Wang Y (2017). Catechol-O-methyltransferase gene promoter methylation as a peripheral biomarker in male schizophrenia. Eur Psychiatry.

[27] Hu TM, Hsu SH, Tsai SM, Cheng MC (2017). DNA methylation analysis of the EGR3 gene in patients of schizophrenia. Psychiatry Res.

[28] Yoshino Y, Ozaki Y, Yamazaki K, Sao T, Mori Y, Ochi S (2017). DNA methylation changes in Intron 1 of triggering receptor expressed on myeloid cell 2 in japanese Schizophrenia subjects. Front Neurosci.

[29] Li Y, Wang KS, Zhang P, Huang J, An H, Wang N (2018). Quantitative DNA methylation analysis of DLGAP2 gene using pyrosequencing in schizophrenia with tardive dyskinesia: a linear mixed model approach. Sci Rep.

[30] Kolble K (1993). Regional mapping of short tandem repeats on human chromosome 10: cytochrome P450 gene CYP2E, D10S196, D10S220, and D10S225. Genomics.

[31] Hayashi S, Watanabe J, Kawajiri K (1991). Genetic polymorphisms in the 5-prime-flanking region change transcriptional regulation of the human cytochrome P450IIE1 gene. J Biochem.

[32] Wang S-M, Zhu A-P, Li D, Wang Z, Zhang P, Zhang G-L (2009). Frequencies of genotypes and alleles of the functional SNPs in CYP2C19 and CYP2E1 in mainland chinese Kazakh, Uygur and Han populations. J Hum Genet.

[33] Catanzaro I, Naselli F, Saverini M, Giacalone A, Montalto G, Caradonna F (2012). Cytochrome P450 2E1 variable number tandem repeat polymorphisms and health risks: a genotype-phenotype study in cancers associated with drinking and/or smoking. Mol Med Rep.

[34] Huo R, Tang K, Wei Z, Shen L, Xiong Y, Wu X (2012). Genetic polymorphisms in CYP2E1: association with schizophrenia susceptibility and risperidone response in the chinese Han population. PLoS ONE.

[35] Kaut O, Schmitt I, Wüllner U (2012). Genome-scale methylation analysis of Parkinson’s disease patients’ brains reveals DNA hypomethylation and increased mRNA expression of cytochrome P450 2E1. Neurogenetics.

[36] Naselli F, Catanzaro I, Bellavia D, Perez A, Sposito L, Caradonna F (2014). Role and importance of polymorphisms with respect to DNA methylation for the expression of CYP2E1 enzyme. Gene.

[37] Kumsta R, Marzi SJ, Viana J, Dempster EL, Crawford B, Rutter M (2016). Severe psychosocial deprivation in early childhood is associated with increased DNA methylation across a region spanning the transcription start site of CYP2E1. Transl Psychiatry.

[38] Chamorro JG, Castagnino JP, Aidar O (2017). Effect of gene-gene and gene-environment interactions associated with antituberculosis drug-induced hepatotoxicity. Pharmacogenet Genomics.

[39] Hartman JH, Miller GP, Meyer JN (2017). Toxicological implications of mitochondrial localization of CYP2E1. Toxicol Res (Camb).

[40] Zhang W, Lu D, Dong W, Zhang L, Zhang X, Quan X (2011). Expression of CYP2E1 increases oxidative stress and induces apoptosis of cardiomyocytes in transgenic mice. FEBS J.

[41] Lakshman MR, Garige M, Gong MA, Leckey L, Varatharajalu R, Redman RS (2013). CYP2E1, oxidative stress, post-translational modifications and lipid metabolism. Subcell Biochem.

[42] Jiménez-Garza O, Baccarelli AA, Byun HM, Márquez-Gamiño S, Barrón-Vivanco BS, Albores A (2015). CYP2E1 epigenetic regulation in chronic, low-level toluene exposure: relationship with oxidative stress and smoking habit. Toxicol Appl Pharmacol.

[43] Schooler NR, Kane JM (1982). Research diagnoses for tardive dyskinesia. Arch Gen Psychiatry.

[44] Kay SR, Fiszbein A, Opler LA (1987). The positive and negative syndrome scale (PANSS) for schizophrenia. Schizophr Bull.

[45] Lin L, Xu C (2020). Arcsine-based transformations for meta-analysis of proportions: pros, cons, and alternatives. Health Sci Rep.

[46] Bolt HM, Roos PH, Their R (2003). The cytochrome P-450 isoenzyme CYP2E1 in the biological processing of industrial chemicals: consequences for occupational and environmental medicine. Int Arch Occup Environ Health.

[47] Gonzalez FJ (2005). Role of cytochromes P450 in chemical toxicity and oxidative stress: studies with CYP2E1. Mutat Res.

[48] Liu H, Baliga R (2003). Cytochrome P450 2E1 null mice provide novel protection against cisplatin-induced nephrotoxicity and apoptosis. Kidney Int.

[49] Halliwell B (1999). Antioxidant defence mechanisms: from the beginning to the end (of the beginning). Free Radic Re.

[50] Zhang H, KJA D, Forman HJ. Oxidative stress response and Nrf2 signaling in aging. Free Radic Biol Med. 2105; 88(Pt B):314–36. 10.1016/j.freeradbiomed.2015.05.036 PMC4628850 26066302

[51] Hackett JA, Surani MA (2013). DNA methylation dynamics during the mammalian life cycle. Philos Trans R Soc Lond B Bio Sci.

[52] Gordon K, Clouaire T, Bao XX, Kemp SE, Xenophontos M, de Las Heras JI (2014). Immortality, but not oncogenic transformation, of primary human cells leads to epigenetic reprogramming of DNA methylation and gene expression. Nucleic Acids Res.

[53] Shah A, Ong CE, Pan Y (2021). Unveiling the role of cytochrome P450 (2E1) in human brain specifically in Parkinson’s Disease - Literature Review. Curr Drug Metab.

[54] Kaut O, Schmitt I, Stahl F, Fröhlich H, Hoffmann P, Gonzalez FJ, Wüllner U (2022). Epigenome-Wide analysis of DNA methylation in Parkinson’s Disease Cortex. Life (Basel).

[55] Iwahashi K, Nakamura K, Furukawa A, Okuyama E, Miyatake R (1997). No linkage of the cytochrome P-450IIE1 (CYP2E1) C1/C2 polymorphism to schizophrenia. Hum Exp Toxicol.

[56] Zhu Y, Mordaunt CE, Yasui DH, Marathe R, Coulson RL, Dunaway KW, Jianu JM, Walker CK, Ozonoff S, Hertz-Picciotto I, Schmidt RJ, LaSalle JM (2019). Placental DNA methylation levels at CYP2E1 and IRS2 are associated with child outcome in a prospective autism study. Hum Mol Genet.

[57] Zhang P, Li YL, An HM, Tan YL (2018). Preliminary construction of DNA methylation profiles of schizophrenia patients with tardive dyskinesia. Chin J Psychiatry.

[58] Braun PR, Han S, Hing B, Nagahama Y, Gaul LN, Heinzman JT (2019). Genome-wide DNA methylation comparison between live human brain and peripheral tissues within individuals. Transl Psychiatry.

[59] High R. An introduction to statistical power calculations for linear models with SAS 9.1. https://www.lexjansen.com/pnwsug/2007/Robin%20High%20-%20Statistical%20Power%20Calculations%20for%20Linear%20Models.pdf (2007). 2007.

